# Multiplex APLP System for High-Resolution Haplogrouping of Extremely Degraded East-Asian Mitochondrial DNAs

**DOI:** 10.1371/journal.pone.0158463

**Published:** 2016-06-29

**Authors:** Tsuneo Kakuda, Hideki Shojo, Mayumi Tanaka, Phrabhakaran Nambiar, Kiyoshi Minaguchi, Kazuo Umetsu, Noboru Adachi

**Affiliations:** 1 Department of Legal Medicine, Interdisciplinary Graduate School of Medicine and Engineering, University of Yamanashi, 1110 Shimokato, Yamanashi 409–3898, Japan; 2 Department of General Dental Practice and Oral & Maxillofacial Imaging, Faculty of Dentistry, University of Malaya, 50603 Kuala Lumpur, Malaysia; 3 Department of Forensic Medicine, Tokai University School of Medicine, 143 Shimokasuya, Kanagawa 259–1193, Japan; 4 Department of Forensic Medicine, Faculty of Medicine, Yamagata University, 2-2-2 Iida-nishi, Yamagata 990–2331, Japan; Waseda University, JAPAN

## Abstract

Mitochondrial DNA (mtDNA) serves as a powerful tool for exploring matrilineal phylogeographic ancestry, as well as for analyzing highly degraded samples, because of its polymorphic nature and high copy numbers per cell. The recent advent of complete mitochondrial genome sequencing has led to improved techniques for phylogenetic analyses based on mtDNA, and many multiplex genotyping methods have been developed for the hierarchical analysis of phylogenetically important mutations. However, few high-resolution multiplex genotyping systems for analyzing East-Asian mtDNA can be applied to extremely degraded samples. Here, we present a multiplex system for analyzing mitochondrial single nucleotide polymorphisms (mtSNPs), which relies on a novel amplified product-length polymorphisms (APLP) method that uses inosine-flapped primers and is specifically designed for the detailed haplogrouping of extremely degraded East-Asian mtDNAs. We used fourteen 6-plex polymerase chain reactions (PCRs) and subsequent electrophoresis to examine 81 haplogroup-defining SNPs and 3 insertion/deletion sites, and we were able to securely assign the studied mtDNAs to relevant haplogroups. Our system requires only 1×10^−13^ g (100 fg) of crude DNA to obtain a full profile. Owing to its small amplicon size (<110 bp), this new APLP system was successfully applied to extremely degraded samples for which direct sequencing of hypervariable segments using mini-primer sets was unsuccessful, and proved to be more robust than conventional APLP analysis. Thus, our new APLP system is effective for retrieving reliable data from extremely degraded East-Asian mtDNAs.

## Introduction

Mitochondrial DNA (mtDNA) is a powerful tool for exploring matrilineal phylogeographic ancestry, as well as for analyzing highly degraded samples, because of its polymorphic nature and high copy numbers per cell. The recent advent of complete mitochondrial genome sequencing has led to improved techniques for phylogenetic analyses based on mtDNA, and many multiplex genotyping methods have been developed for the hierarchical analysis of phylogenetically important mutations [1–7].

However, few multiplex genotyping systems for analyzing East-Asian mtDNA lineage can be applied to extremely degraded samples [2, 5–7]. Even in these studies, haplogroup D, which exhibits the highest frequency and incidence of variations in many East-Asian populations, is not sufficiently classified. For example, Coutinho et al. [6] divided haplogroup D into 8 sub-haplogroups (the highest number among the above-mentioned studies). However, with the exception of sub-haplogroups D4b1 and D4e, these sub-haplogroups are exclusively observed in Native Americans; moreover, many sub-haplogroups of haplogroup D that are phylogenetically important in East-Asian populations are missing (e.g., haplogroup D4a). Therefore, there is a need to establish higher resolution multiplex systems for the hierarchical analysis of phylogenetically important mutations in East-Asian populations.

Among the methods for analysis of single nucleotide polymorphisms in mtDNA (mtSNPs), amplified product-length polymorphism (APLP) [8, 9] is considered one of the simplest and most robust. To detect mtSNPs, APLP employs two allele-specific primers, one of which has a few non-complementary bases in the 5'-terminus. The detection consists of assessing the difference in the length of the amplicons, which are obtained by polymerase chain reaction (PCR) and subsequent electrophoresis. We previously showed the effectiveness of APLP-based multiplex mtSNP analyses [9] for highly degraded samples when we successfully clarified the genealogy of individuals, and the relationship between populations excavated from different archaeological sites [10–16].

However, with respect to the successful analysis of extremely degraded samples, the conventional mitochondrial APLP (mtAPLP) system [9] has at least four drawbacks. First, conventional mtAPLP systems examine 35 haplogroup-diagnostic mtSNPs and a 9-bp repeat variation in the non-coding cytochrome oxidase II/tRNA^Lys^ intergenic region. This number of polymorphic sites is too small for classifying mtDNAs to sub-haplogroup level without using the sequence data of the hypervariable segments (HVS). Second, in each set of a conventional mtAPLP system, the mtSNPs are not selected in accordance with the phylogenetic order. For instance, the macro-haplogroup examined in set A is N despite the fact that seven out of nine haplogroups examined in this set stem from macro-haplogroup M: haplogroup D, its branches (D4, D4a, D4b, D4g, and D4e), and haplogroup M12. Third, the competitiveness of some primers is low. For example, haplogroup F mtDNA always shows an extra 66-bp band on gel. Fourth, the amplicon size is considered inappropriate. In practice, amplicons longer than 120 bp frequently disappear when analyzing extremely degraded samples. To overcome such limitations, a more accurate, detailed, and sensitive mtDNA haplogrouping system is required.

Here, we present a novel multiplex inosine-flapped APLP system that is specifically designed for haplogrouping extremely degraded East-Asian mtDNAs.

## Materials and Methods

### DNA samples

To obtain modern-day DNA samples, intraoral epithelial cells were collected from eight healthy Japanese adults. Before cells were collected, volunteers were informed, in writing that their DNA would be anonymized and that it would be used only for haplogrouping of its mtDNA. Written consent was then obtained from each volunteer to use his or her DNA in the study. Both the consent procedure and, the written forms, were approved by the ethics committee of the Faculty of Medicine of the University of Yamanashi.

In order to establish the current APLP system, in addition to the samples from the volunteers, we also used ancient and modern-day DNA samples for which the mtDNA haplogroups had been securely determined in previous studies [9–17]. DNA samples provided by the University of Malaya, Tokai University School of Medicine, and the Yamagata University had all been anonymized before arriving at our research facility at the University of Yamanashi. We obtained permission to conduct this study using these DNA samples from each of the respective universities.

The intraoral epithelial cells were collected using a forensic swab (Sarstedt Inc., Nümbrecht, Germany). DNA extraction was performed using a MonoFas^®^ Intraoral epithelial cells genome DNA extraction kit VIII (GL Science Inc., Tokyo, Japan), and the manufacturer’s protocol was followed. The quantity and purity of the DNA was evaluated by optical density (OD)_260_ and OD_260/280_ measurements, obtained using a spectrophotometer (Nano Drop 1000; Thermo Fisher Scientific Inc., Waltham, MA, USA).

**Fig 1:**
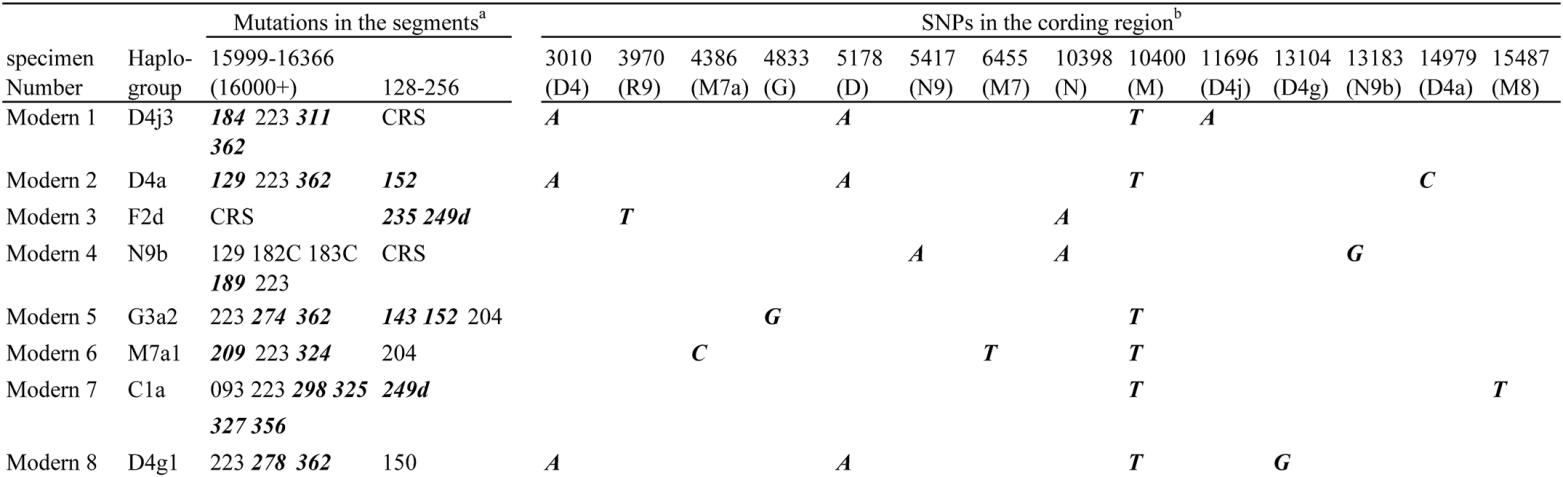
Nucleotide changes observed in modern-day DNA samples. ^a^All polymorphic sites are numbered according to the rCRS [18]. CRS denotes that the sequence of the segment is identical to the rCRS. The suffix A indicates a transversion, and d indicates a deletion. Deletions are recorded at the last possible site. Diagnostic polymorphisms are emphasized by bold italic type. ^b^All polymorphic sites are numbered according to the rCRS. Diagnostic polymorphisms are emphasized by bold italic type. The sites that did not show polymorphisms are omitted.

To determine the mtDNA haplogroups from these DNA samples, segments of mtDNA that cover parts of the tRNA^Pro^ gene, the hypervariable segments (HVS) 1 (nucleotide position (np) 15999–16366, relative to the revised Cambridge reference sequence (rCRS) [18]), and HVS 2 (np 128–256) were analyzed as described previously [12]. Moreover, to confirm our ability to identify mtDNA haplogroups from modern-day samples, we also analyzed haplogroup-diagnostic mtSNPs and a 9-bp repeat variation in the non-coding cytochrome oxidase II/tRNA^Lys^ intergenic region by using the conventional mtAPLP system [9]. Nucleotide changes observed in eight modern-day samples are shown in Fig 1. Thereafter, we assigned each modern-day mtDNA under study to the relevant haplogroup by using Phylotree, the updated comprehensive phylogenetic tree of global human mitochondrial DNA variation (www.phylotree.org; mtDNA tree Build 17) [19]. Basically, Phylotree is built based on the Reconstructed Sapience Reference Sequence (RSRS) [20] to avoid inconsistencies, misinterpretations, and errors in medical, forensic, and population genetic studies. However, the description of nucleotide changes in the conventional mtAPLP system [9] is based on rCRS. Therefore, we used an rCRS-oriented version of mtDNA tree Build 17 [19] as a classification tree for the modern-day samples.

Furthermore, in order to evaluate the effectiveness of our system for the analysis of extremely degraded samples, we also tested ancient DNA samples extracted from one early Kofun (approximately 1,600 years old) and 11 Middle Jomon (approximately 4,000 years old) skeletons excavated from the Kusakari shell midden site, Chiba, Japan. DNA was extracted from the teeth of these skeletons according to the method described by Adachi et al. [14].

### Hierarchical analysis of phylogenetically important East-Asian mtSNPs

**Fig 2:**
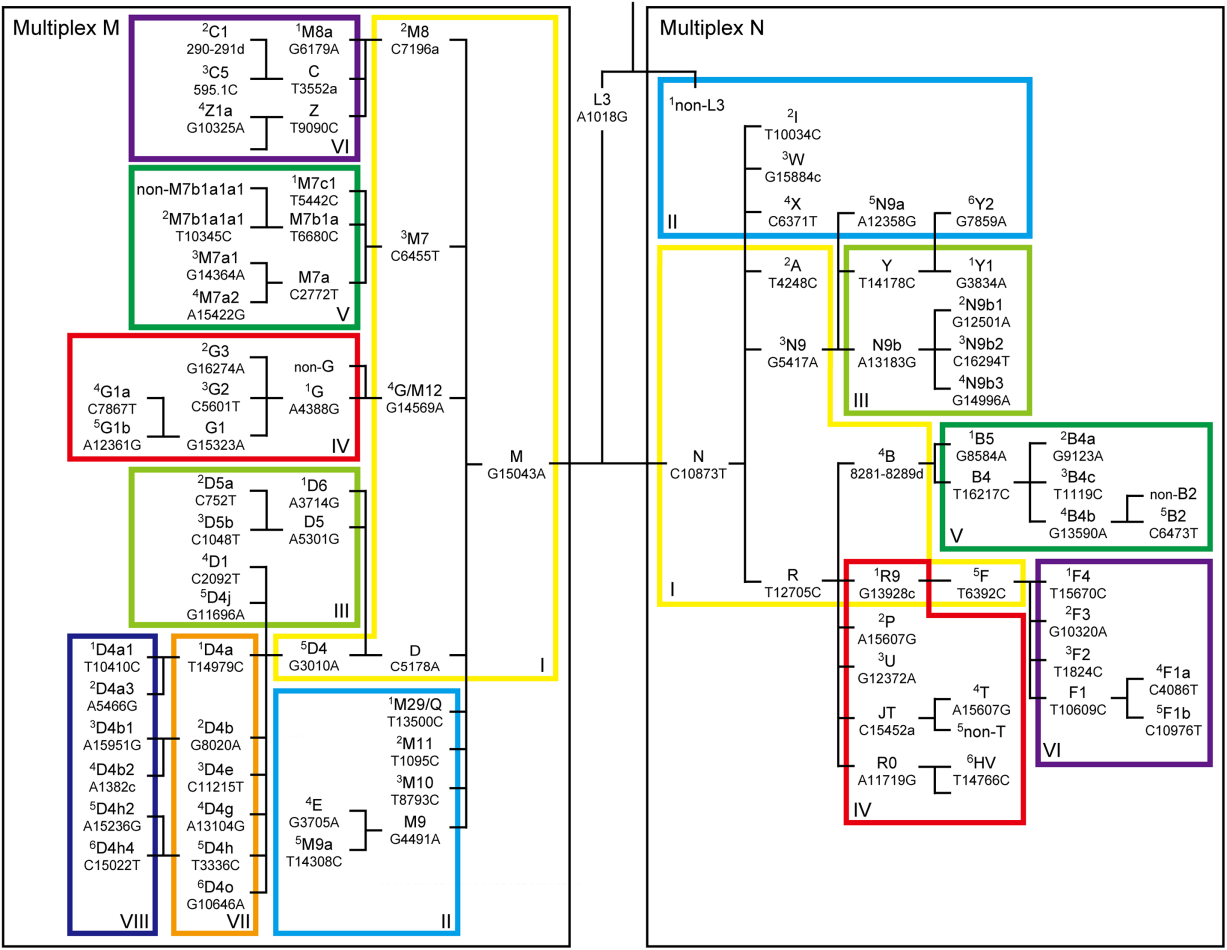
Scheme of mitochondrial DNA haplogroup assignment based on the haplogroup-defining mutations. The color coding of the frames is consistent with that employed to identify electrophoretograms in Figs 3 and 4. Superscript numbers correspond to the electrophoretogram lane numbers shown in Figs 3 and 4. The primer sets of multiplexes M-I to M-VIII and N-I to N-VI are shown in Tables 1 and 2, respectively.

To securely assign East-Asian mtDNAs to the relevant haplogroups, we analyzed 81 haplogroup-diagnostic SNPs, and three deletion/insertion polymorphisms (Fig 2): for haplogroup B, a 9-bp deletion in the non-coding cytochrome oxidase II/tRNA^Lys^ intergenic region; for haplogroup C5, a cytosine insertion just after nucleotide position 595 (595.1 C); for haplogroup C1, a 2-bp deletion at positions 290–291. Each nucleotide position is numbered according to the RSRS [20]. These mutation sites were selected based on the RSRS-oriented version of mtDNA tree Build 17 [19], in consideration of the frequency and significance of the haplogroups observed in modern-day and ancient East Asian populations, as suggested by previous studies [9, 13–17, 21–27].

**Fig 3:**
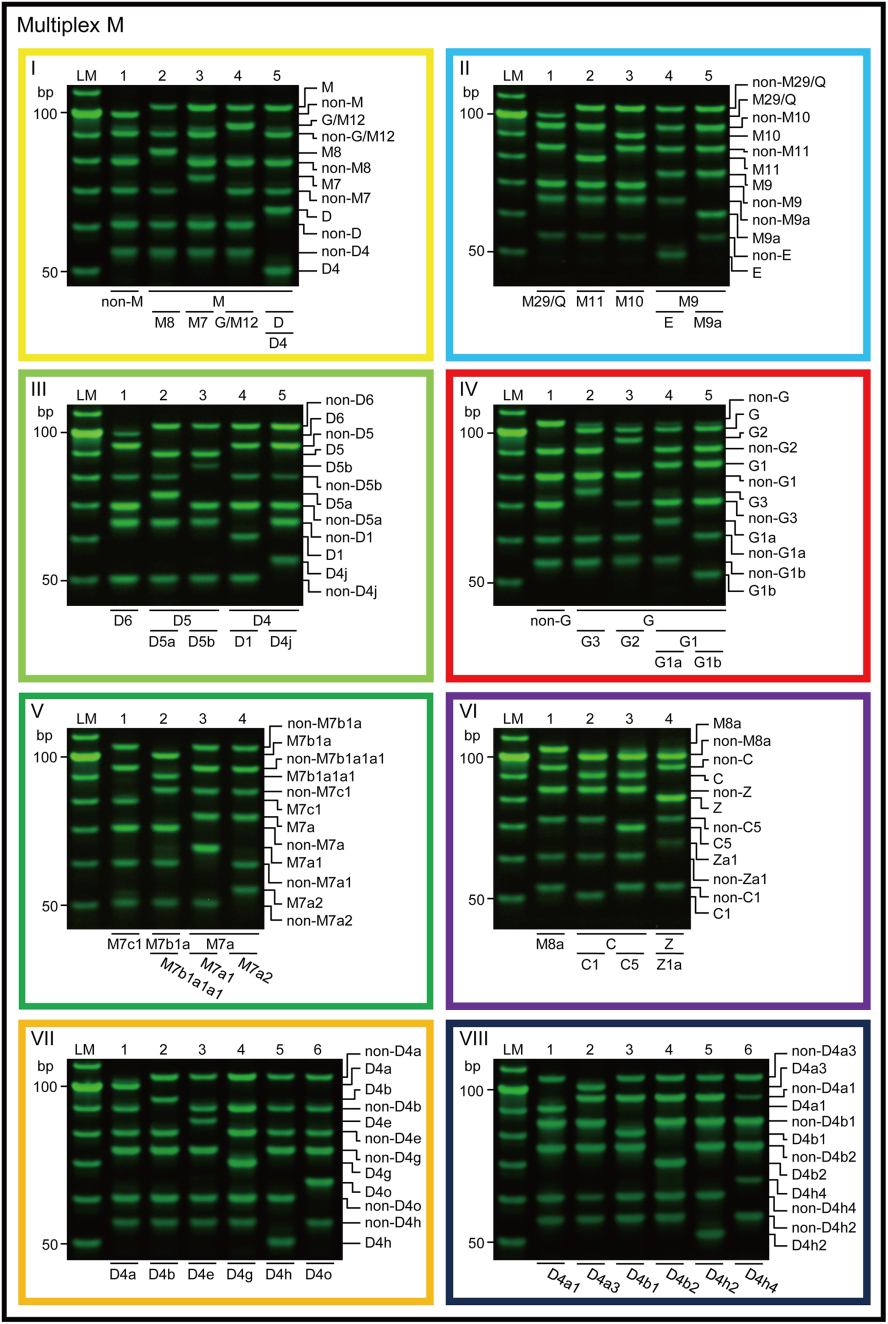
Electrophoretogram of PCR products from multiplexes M-I to M-VIII. The primer sets of multiplexes M-I to M-VIII are shown in Table 2. Yellow, light blue, light green, red, green, purple, orange, and blue frames indicate multiplexes M-I, II, III, IV, V, VI, VII, and VIII, respectively. This color coding corresponds to that given in Fig 2. LM indicates the 10-bp ladder marker.

**Fig 4:**
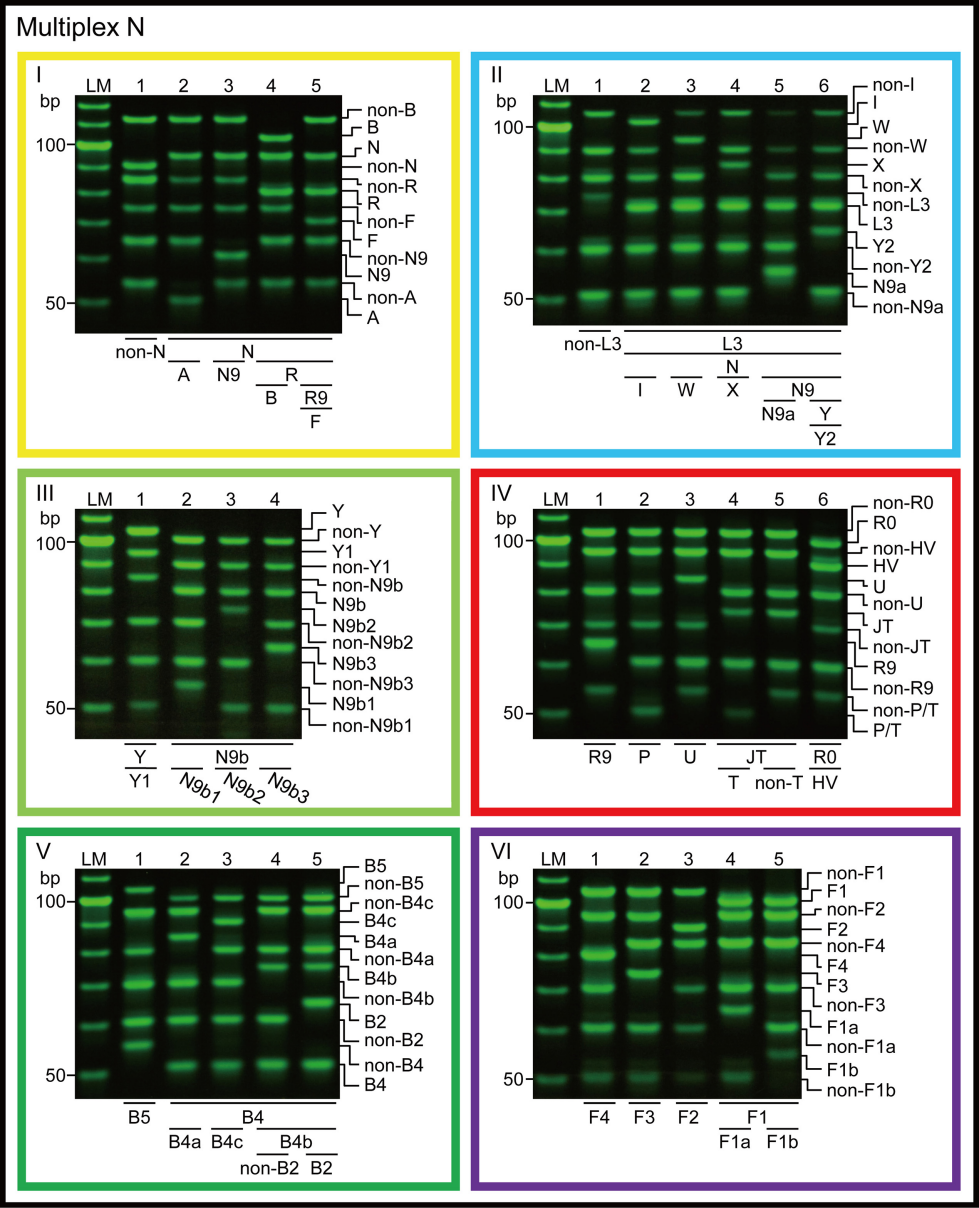
Electrophoretogram of PCR products from multiplexes N-I to N-VI. The primer sets of multiplexes N-I to N-VI are shown in Table 2.Yellow, light blue, light green, red, green, and purple frames indicate multiplexes N-I, II, III, IV, V, and VI, respectively. The color coding corresponds to that given in Fig 2. LM indicates the 10-bp ladder marker.

Phylogenetically important mutations were examined using fourteen 6-plex PCR sets (Table 1 and Fig 3; Table 2 and Fig 4). We recently developed novel APLP primers with a short inosine extension added to the 5′-terminus. This modification improves the competitiveness of allele-specific primers to the template DNA, resulting in enhanced reliability of the analysis of SNPs [28]. In the present study, we designed the primers based on this inosine-flapped APLP method. Moreover, to maximize the robustness of the PCR, we used amplicons with a length of <110 bp, which is shorter than the amplicon length used in the conventional system (<151 bp) [9]; we also examined fewer polymorphic sites in each set (6 sites, compared to 9 in the conventional system) [9].

**Table 1:** Primers of multiplexes M-I to M-VIII used for haplogrouping mtDNAs that stem from macro-haplogroup M.

**Multiplex M-I**				
**Haplogroup**	**Primer name**	**Sequence**	**Concentration (μM)**	**Amplicon length (bp)**
M	M-15043A	IIII ATC CGT AAT ATA GGC CTC G***T***	0.2	104
non-M	M-15043G	ATC CGT AAT ATA GGC CTC G***C***	0.2	100
	M-15043F	GTA AAT TAT GGC TGA ATC ATC CGC	0.2	
G/M12	G/M12- 14569A	II TAA CAC ACC CGA CCA CAC C***A***	0.2	94
non-G/M12	G/M12- 14569G	ACA CAC CCG ACC ACA CC***G***	0.2	90
	G/M12- 14569R	TTT AGT AAT GGG GTT TGT GGG GT	0.2	
M8	M8-7196A	II AAC TTT CTT CCC ACA ACA CTT TCT ***A***	0.2	84
non-M8	M8-7196C	CTT TCT TCC CAC AAC ACT TTC T***C***	0.2	80
	M8-7196R	TCA TGT GGT GTA TGC ATC GGG	0.2	
M7	M7-6455T	IIII CAA TAC CAA ACG CCC CTC TT***T***	0.1	74
non-M7	M7-6455C	CAA TAC CAA ACG CCC CTC TT***C***	0.2	70
	M7-6455R	CTG GGA GAG ATA GGA GAA GTA G	0.2	
D	D-5178A	III CTA TCT CGC ACC TGA AAC AAG ***A***	0.2	64
non-D	D-5178C	TAT CTC GCA CCT GAA ACA AG***C***	0.2	60
	D-5178R	AGA GGA GGG TGG ATG GAA TTA A	0.2	
non-D4	D4-3010G	III TAA TAG CGG CTG CAC CAT ***C***	0.1	54
D4	D4-3010A	AAT AGC GGC TGC ACC AT***T***	0.2	50
	D4-3010F	TTT ACG ACC TCG ATG TTG GAT C	0.2	
**Multiplex M-II**				
**Haplogroup**	**Primer name**	**Sequence**	**Concentration (μM)**	**Amplicon length (bp)**
non-M29/Q	M29/Q-13500T	III GGA ATA CCT TTC CTC ACA GG***T***	0.2	104
M29/Q	M29/Q-13500C	GAA TAC CTT TCC TCA CAG G***C***	0.2	100
	M29/Q-13500R	GCG ATG AGA GTA ATA GAT AGG G	0.2	
non-M10	M10-8793T	IIII GTG GTT GGT GTA AAT GAG TG***A***	0.2	94
M10	M10-8793C	GTG GTT GGT GTA AAT GAG TG***G***	0.1	90
	M10-8793F	AAC CTG ATC TCT TAT ACT AGT ATC CTT AAT C	0.2	
non-M11	M11-1095T	III GGA TTA GAT ACC CCA CTA TGC T***T***	0.1	84
M11	M11-1095C	GAT TAG ATA CCC CAC TAT GCT ***C***	0.2	80
	M11-1095R	GTG GCT CGT AGT GTT CTG G	0.2	
M9	M9-4491A	IIII GCA AAG ATG GTA GAG TAG ATG A***T***	0.2	74
non-M9	M9-4491G	GCA AAG ATG GTA GAG TAG ATG A***C***	0.2	70
	M9-4491F	ATG TTG GTT ATA CCC TTC CCG T	0.2	
non-M9a	M9a-14308T	IIII GTT GTG GTA AAC TTT AAT AGT GTA GG***A***	0.2	64
M9a	M9a-14308C	GTT GTG GTA AAC TTT AAT AGT GTA GG***G***	0.2	60
	M9a-14308F	CCC TGA CCC CTC TCC TTC A	0.2	
non-E	E-3705G	III TGT TTG GGC TAC TGC TCG ***C***	0.05	54
E	E-3705A	GTT TGG GCT ACT GCT CG***T***	0.2	50
	E-3705F	TCA AAC TCA AAC TAC GCC CTG A	0.2	
**Multiplex M-III**				
**Haplogroup**	**Primer name**	**Sequence**	**Concentration (μM)**	**Amplicon length (bp)**
non-D6	D6-3714A	III CTT CAT ATG AGA TTG TTT GGG C***T***	0.2	104
D6	D6-3714G	TTC ATA TGA GAT TGT TTG GGC ***C***	0.2	100
	D6-3714F	CCT AGC CGT TTA CTC AAT CCT	0.2	
non-D5	D5-5301A	II TTC ACA AAA AAC AAT AGC CTC ATC ***A***	0.2	94
D5	D5-5301G	CAC AAA AAA CAA TAG CCT CAT C***G***	0.2	90
	D5-5301R	GTG GAG TAG ATT AGG CGT AGG	0.2	
D5b	D5b -1048T	III GTT TGG GTC TTA GCT ATT GTG T***A***	0.1	84
non-D5b	D5b -1048C	TTT GGG TCT TAG CTA TTG TGT ***G***	0.2	80
	D5b -1048F	TGT AAA AAA CTC CAG TTG ACA CAA A	0.2	
D5a	D5a -752T	III AAT CAC CAC GAT CAA AAG GGA ***T***	0.2	74
non-D5a	D5a -752C	ATC ACC ACG ATC AAA AGG GA***C***	0.2	70
	D5a -752R	TGT GGC TAG GCT AAG CGT TTT	0.2	
non-D1	D1-2092C	IIII GCT GTT CCT CTT TGG ACT AAC A***G***	0.2	64
D1	D1-2092T	GCT GTT CCT CTT TGG ACT AAC A***A***	0.2	60
	D1-2092F	TGC CCA CAG AAC CCT CTA AAT C	0.2	
D4j	D4j-11696A	III CGT GGG CGA TTA TGA GAA TGA ***T***	0.1	54
non-D4j	D4j-11696G	GTG GGC GAT TAT GAG AAT GA***C***	0.2	50
	D4j-11696F	TCC AAA CCC CCT GAA GCT TC	0.2	
**Multiplex M-IV**				
**Haplogroup**	**Primer name**	**Sequence**	**Concentration (μM)**	**Amplicon length (bp)**
non-G	G-4833A	IIII CCA GAG GTT ACC CAA GGC ***A***	0.2	104
G	G-4833G	CCA GAG GTT ACC CAA GGC ***G***	0.2	100
	G-4833R	GTG AGG GAG AGA TTT GGT ATA TG	0.2	
G2	G2-5601T	IIII TAA CAG CTA AGG ACT GCA AAA ***T***	0.2	94
non-G2	G2-5601C	TAA CAG CTA AGG ACT GCA AAA ***C***	0.2	90
	G2-5601R	CCC ATT GGT CTA GTA AGG GC	0.2	
G1	G1-15323A	III AAT AGG AGG TGG AGT GCT G***T***	0.2	84
non-G1	G1-15323G	ATA GGA GGT GGA GTG CTG ***C***	0.2	80
	G1-15323F	TCC CAC CCT CAC ACG ATT CT	0.2	
G3	G3 -16274A	III GGG TGG GTA GGT TTG TTG RTA T***T***	0.1	74
non-G3	G3 -16274G	GGT GGG TAG GTT TGT TGR TAT ***C***	0.2	70
	G3 -16274F	AAC TAT CAC ACA TCA ACT GCA ACT	0.2	
G1a	G1a-7867T	III GGC CAA TTG ATT TGA TGG TAA G***A***	0.2	64
non-G1a	G1a-7867C	GCC AAT TGA TTT GAT GGT AAG ***G***	0.2	60
	G1a-7867F	CGC ATC CTT TAC ATA ACA GAC G	0.2	
non-G1b	G1b-12361A	II TAA CCA TGC ACA CTA CTA TAA CC***A***	0.2	54
G1b	G1b-12361G	ACC ATG CAC ACT ACT ATA ACC ***G***	0.2	50
	G1b-12361R	GGG GGA ATT AGG GAA GTC AG	0.2	
**Multiplex M-V**				
**Haplogroup**	**Primer name**	**Sequence**	**Concentration (μM)**	**Amplicon length (bp)**
non-M7b1a	M7b1a -6680T	III GGT TCT TTT TTT CCG GAG TAG TA***A***	0.1	104
M7b1a	M7b1a -6680C	GTT CTT TTT TTC CGG AGT AGT A***G***	0.2	100
	M7b1a -6680F	CTA TTC TGA TTT TTC GGT CAC CC	0.2	
non- M7b1a1a1	M7b1a1a1-10345T	III CAG ACT TAG GGC TAG GAT G***A***	0.1	94
M7b1a1a1	M7b1a1a1-10345C	AGA CTT AGG GCT AGG ATG ***G***	0.2	90
	M7b1a1a1-10345F	TTT ACC CCT ACC ATG AGC CC	0.2	
non-M7c1	M7c1-5442T	III TGA ACA TAC AAA ACC CAC CCC A***T***	0.1	84
M7c1	M7c1-5442C	GAA CAT ACA AAA CCC ACC CCA ***C***	0.2	80
	M7c-5442R	AGT ATA AAA GGG GAG ATA GGT AGG	0.2	
M7a	M7a-2772T	IIII GCA AAC AGT ACC TAA CAA ACC ***T***	0.1	74
non-M7a	M7a-2772C	GCA AAC AGT ACC TAA CAA ACC ***C***	0.2	70
	M7a-2772R	TCG CCC CAA CCG AAA TTT TTA AT	0.2	
M7a1	M7a1-14364A	III CAT CAT ACT CTT TCA CCC ACA ***A***	0.2	64
non-M7a1	M7a1-14364G	ATC ATA CTC TTT CAC CCA CA***G***	0.2	60
	M7a1-14364R	AGT GTT TTA GTG GGG TTA GCG	0.2	
M7a2	M7a2-15422G	IIII CCG AGG GCG TCT TTG A***C***	0.1	54
non-M7a2	M7a2-15422A	I CCG AGG GCG TCT TTG A***T***	0.2	50
	M7a2-15422F	CCG ATA AAA TCA CCT TCC ACC C	0.1	
**Multiplex M-VI**				
**Haplogroup**	**Primer name**	**Sequence**	**Concentration (μM)**	**Amplicon length (bp)**
M8a	M8a-6179A	I TAA TAA TCG GTG CCC CCG ATA T***A***	0.1	104
non-M8a	M8a-6179G	TAA TCG GTG CCC CCG ATA T***G***	0.2	100
	M8a-6179R	TCC ACT ATA GCA GAT GCG AGC	0.2	
non-C	C-3552T	III ACC TTA GCT CTC ACC ATC GC***T***	0.1	94
C	C-3552A	CCT TAG CTC TCA CCA TCG C***A***	0.2	90
	C-3552R	GAA TAA ATA GGA GGC CTA GGT TGA	0.2	
non-Z	Z-9090T	III AAT ATC AAC CAT TAA CCT TCC CTC ***T***	0.2	84
Z	Z-9090C	ATA TCA ACC ATT AAC CTT CCC TC***C***	0.2	80
	Z-9090R	GCG ACA GCG ATT TCT AGG ATA G	0.2	
non-C5	C5-W/O 595.1C	III ACA GTT TAT GTA GCT TAC CTC C***T***	0.1	74
C5	C5-595.1C	CAG TTT ATG TAG CTT ACC TCC ***C***	0.2	70
	C5-595.1C-R	ATG GGG TGA TGT GAG CCC	0.2	
Z1a	Z1a-10325A	III AAC TAA CCT GCC ACT AAT AGT TAT ***A***	0.2	64
non-Z1a	Z1a-10325G	ACT AAC CTG CCA CTA ATA GTT AT***G***	0.2	60
	Z1a-10325R	ACT TAG GGC TAG GAT GAT GAT TAA TAA	0.2	
C1 or non-C1	290-291d-F	AGC CGC TTT CCA CAC AGA CA	0.2	52 (non-C1) 50 (C1)
	290-291d-R	AGG GGG GGT TTG GTG GAA ATT	0.2	
**Multiplex M-VII**				
**Haplogroup**	**Primer name**	**Sequence**	**Concentration (μM)**	**Amplicon length (bp)**
non-D4a	D4a-14979T	III GCG TGA AGG TAG CGG ATG ***A***	0.1	104
D4a	D4a-14979C	CGT GAA GGT AGC GGA TG***G***	0.2	100
	D4a-14979F	CTA GCC ATG CAC TAC TCA CC	0.2	
D4b	D4b-8020A	II TTA TAC GAA TGG GGG CTT CAA T***T***	0.1	94
non-D4b	D4b-8020G	ATA CGA ATG GGG GCT TCA AT***C***	0.2	90
	D4b-8020F	ACT TCC CCC ATT ATT CCT AGA AC	0.2	
D4e	D4e-11215T	IIII CAG GCA CAT ACT TCC TAT TCT A***T***	0.1	84
non-D4e	D4e-11215C	CAG GCA CAT ACT TCC TAT TCT A***C***	0.2	80
	D4e-11215R	TAG GGT GTT GTG AGT GTA AAT TAG	0.2	
non-D4g	D4g-13104A	III CAA GCA CTA TAG TTG TAG CAG G***A***	0.2	74
D4g	D4g-13104G	AAG CAC TAT AGT TGT AGC AGG ***G***	0.2	70
	D4g-13104R	TAG TGG GCT ATT TTC TGC TAG G	0.2	
D4o	D4o-10646A	III CAC TCC CTC TTA GCC AAT ATT GT***A***	0.2	64
non-D4o	D4o-10646G	ACT CCC TCT TAG CCA ATA TTG T***G***	0.2	60
	D4o-10646R	CTG CTT CGC AGG CGG CAA A	0.2	
non-D4h	D4h-3336T	IIII CCA ACC TCC TAC TCC TCA T***T***	0.1	54
D4h	D4h-3336C	CCA ACC TCC TAC TCC TCA T***C***	0.2	50
	D4h-3336R	TAG GAA TGC CAT TGC GAT TAG AAT	0.2	
**Multiplex M-VIII**				
**Haplogroup**	**Primer name**	**Sequence**	**Concentration (μM)**	**Amplicon length (bp)**
non-D4a3	D4a3-5466A	III ATA GGT AGG AGT AGC GTG G***T***	0.1	104
D4a3	D4a3-5466G	TAG GTA GGA GTA GCG TGG ***C***	0.2	100
	D4a3-5466F	CCC ATA TCT AAC AAC GTA AAA ATA AAA TG	0.2	
non-D4a1	D4a1-10410T	III GAT TAG ACT GAG CTG AAT TGG TA***T***	0.2	94
D4a1	D4a1-10410C	ATT AGA CTG AGC TGA ATT GGT A***C***	0.2	90
	D4a1-10410R	AGG GGC ATT TGG TAA ATA TGA TTA TC	0.2	
non-D4b1	D4b1-15951A	III AGA TGA AAA CCT TTT TCC AAG G***A***	0.2	84
D4b1	D4b1-15951G	GAT GAA AAC CTT TTT CCA AGG ***G***	0.1	80
	D4b1-15951R	AAT TAG AAT CTT AGC TTT GGG TGC T	0.2	
non-D4b2	D4b2-1382A	III GGC TAC ATT TTC TAC CCC AGA ***A***	0.2	74
D4b2	D4b2-1382C	GCT ACA TTT TCT ACC CCA GA***C***	0.2	70
	D4b2-1382R	CTG CTA AAT CCA CCT TCG ACC	0.2	
D4h4	D4h4-15022T	III CAA TGG CGC CTC AAT ATT CTT TAT ***T***	0.2	64
non-D4h4	D4h4-15022C	AAT GGC GCC TCA ATA TTC TTT AT***C***	0.2	60
	D4h4-15022R	GTA ATA TAG GCC TCG CCC GA	0.2	
non-D4h2	D4h2-15236A	II TAC TGA GTA GCC TCC TCA GA***T***	0.1	54
D4h2	D4h2-15236G	CTG AGT AGC CTC CTC AGA ***C***	0.2	50
	D4h2-15236F	CCC ATA CAT TGG GAC AGA CC	0.2	

The letter ‘I’ stands for inosine. Underlined letters indicate non-complementary bases. SNP sites are indicated in bold italics. Haplogroup C1 is detected by the 2-bp deletion at positions 290–291.

**Table 2:** Primers of multiplexes N-I to N-VI used for haplogrouping mtDNAs that stem from macro-haplogroup N.

**Multiplex N-I**				
**Haplogroup**	**Primer name**	**Sequence**	**Concentration (μM)**	**Amplicon length (bp)**
B or non-B	9 bp-F	TGC CCA TCG TCC TAG AAT TAA TTC	0.2	110 (non-B) 101 (B)
	9 bp-R	GCT AAG TTA GCT TTA CAG TGG GC	0.2	
N	N-10873T	III AGC CTA ATT ATT AGC ATC ATC CC***T***	0.2	94
non-N	N-10873C	GCC TAA TTA TTA GCA TCA TCC C***C***	0.2	90
	N-10873R	GGG GGT CGG AGG AAA AGG T	0.2	
non-R	R-12705T	IIII GGT AAC TAA GAT TAG TAT GGT AAT TAG GAA ***A***	0.2	84
R	R-12705C	GGT AAC TAA GAT TAG TAT GGT AAT TAG GAA ***G***	0.2	80
	R-12705F	ATA TAA ACT CAG ACC CAA ACA TTA ATC AGT	0.2	
non-F	F-6392T	III CTC TAT CTT AGG GGC CAT CAA ***T***	0.1	74
F	F-6392C	TCT ATC TTA GGG GCC ATC AA***C***	0.2	70
	F-6392R	GGT ATT GGG TTA TGG CAG GG	0.2	
non-N9	N9-5417G	IIIII CAT ATC TAA CAA CGT AAA AAT AAA ATG ACA ***G***	0.2	64
N9	N9-5417A	CCA TAT CTA ACA ACG TAA AAA TAA AAT GAC A***A***	0.2	60
	N9-5417R	GGA ATG GGG TGG GTT TTG TAT G	0.2	
non-A	A-4248T	IIII ACC CAT TAC AAT CTC CAG CAT ***T***	0.2	54
A	A-4248C	ACC CAT TAC AAT CTC CAG CAT ***C***	0.2	50
	A-4248R	TCA GAC ATA TTT CTT AGG TTT GAG G	0.2	
**Multiplex N-II**				
**Haplogroup**	**Primer name**	**Sequence**	**Concentration (μM)**	**Amplicon length (bp)**
non-I	I-10034T	II TAC CGT TAA CTT CCA ATT AAC TAG ***T***	0.2	104
I	I-10034C	CCG TTA ACT TCC AAT TAA CTA G***C***	0.2	100
	I-10034R	ATT AGT AGT AAG GCT AGG AGG GT	0.2	
W	W-15884C	III ATT AGT TTA TAC TAC AAG GAC AGG ***G***	0.2	94
non-W	W-15884G	TTA GTT TAT ACT ACA AGG ACA GG***C***	0.2	90
	W-15884F	TAC TTC ACA ACA ATC CTA ATC CTA ATA	0.2	
X	X-6371T	III TTA CAC CTA GCA GGT GTC TC***T***	0.18	84
non-X	X-6371C	TAC ACC TAG CAG GTG TCT C***C***	0.2	80
	X-6371R	TAT GGC AGG GGG TTT TAT ATT GAT	0.2	
non-L3	L3-1018A	II ATA TGT TAA AGC CAC TTT CGT AGT ***T***	0.1	74
L3	L3-1018G	ATG TTA AAG CCA CTT TCG TAG T***C***	0.2	70
	L3-1018F	AGC TAA AAC TCA CCT GAG TTG TAA	0.2	
Y2	Y2-7859A	II TAC ATA ACA GAC GAG GTC AAC ***A***	0.1	64
non-Y2	Y2-7859G	CAT AAC AGA CGA GGT CAA C***G***	0.2	60
	Y2-7859R	TAC CAT TGG TGG CCA ATT GAT TTG	0.2	
N9a	N9a-12358G	IIII GAA GTT AGG GTT AGG GTG G***C***	0.2	54
non-N9a	N9a-12358A	GAA GTC AGG GTT AGG GTG G***T***	0.2	50
	N9a-12358F	ATA AAA GTA ATA ACC ATG CAC ACT ACT AT	0.2	
**Multiplex N-III**				
**Haplogroup**	**Primer name**	**Sequence**	**Concentration (μM)**	**Amplicon length (bp)**
Y	Y-14178C	IIIII CCG AGC AAT CTC AAT TAC AA***C***	0.2	104
non-Y	Y-14178T	CCC GAG CAA TCT CAA TTA CAA ***T***	0.2	100
	Y-14178R	ATT GGT GCG GGG GCT TTG TAT	0.2	
Y1	Y1-3834A	III AGA ACA CCT CTG ATT ACT CCT ***A***	0.2	94
non-Y1	Y1-3834G	GAA CAC CTC TGA TTA CTC CT***G***	0.2	90
	Y1-3834R	GTC GAA GGG GGT TCG GTT	0.2	
non-N9b	N9b -13183A	III ACA CTA TGC TTA GGC GCT ***A***	0.1	84
N9b	N9b -13183G	CAC TAT GCT TAG GCG CT***G***	0.2	80
	N9b -13183R	GGC TAC GAT TTT TTT GAT GTC ATT TT	0.2	
N9b2	N9b2-16294T	IIII ATA CCA ACA AAC CTA CCC A***T***	0.2	74
non-N9b2	N9b2-16294C	ATA CCA ACA AAC CTA CCC A***C***	0.2	70
	N9b2-16294R	GTA ATG TGC TAT GTA CGG TAA ATG	0.2	
N9b3	N9b3-14996A	II TGA ATC ATC CGC TAC CTT CAC ***A***	0.1	64
non-N9b3	N9b3-14996G	AAT CAT CCG CTA CCT TCA C***G***	0.2	60
	N9b3-14996R	TGT AGG AAG AGG CAG ATA AAG AAT A	0.2	
N9b1	N9b1-12501A	III CTC TTC CCC ACA ACA ATA TTC AT***A***	0.1	54
non-N9b1	N9b1-12501G	TCT TCC CCA CAA CAA TAT TCA T***G***	0.2	50
	N9b1-12501R	CGA GAT AAT AAC TTC TTG GTC TAG G	0.2	
**Multiplex N-IV**				
**Haplogroup**	**Primer name**	**Sequence**	**Concentration (μM)**	**Amplicon length (bp)**
non-R0	R0-11719A	II ATT CTC ATA ATC GCC CAC GG***A***	0.2	104
R0	R0-11719G	TCT CAT AAT CGC CCA CGG ***G***	0.2	100
	R0-11719R	TCC TTG AGA GAG GAT TAT GAT GC	0.2	
non-HV	HV-14766T	IIII CAA TGA CCC CAA TAC GCA AAA ***T***	0.2	94
HV	HV-14766C	CAA TGA CCC CAA TAC GCA AAA ***C***	0.2	90
	HV-14766R	ATG CGG AGA TGT TGG ATG GG	0.2	
U	U-12372A	III CTA CTA TAA CCA CCC TAA CCC T***A***	0.1	84
non-U	U-12372G	TAC TAT AAC CAC CCT AAC CCT ***G***	0.2	80
	U-12372R	ATG AGT TTT TTT TGT TAG GGT TAA CGA	0.2	
JT	JT-15452A	IIII CCT CGG CTT ACT TCT CTT C***A***	0.2	74
non-JT	JT-15452C	CCT CGG CTT ACT TCT CTT C***C***	0.2	70
	JT-15452R	GTC GCC TAG GAG GTC TGG	0.2	
R9	R9-13928C	IIII ACA TAC TCG GAT TCT ACC CTA ***C***	0.2	64
non-R9	R9-13928G	ACA TAC TCG GAT TCT ACC CTA ***G***	0.2	60
	R9-13928R	TAA GAA GGC CTA GAT AGG GGA T	0.2	
non-P/T	P/T-15607A	III TCC GAT CCG TCC CTA ACA A***A***	0.1	54
P/T	P/T-15607G	CCG ATC CGT CCC TAA CAA ***G***	0.2	50
	P/T-15607R	TGG ATA GTA ATA GGG CAA GGA C	0.2	
**Multiplex N-V**				
**Haplogroup**	**Primer name**	**Sequence**	**Concentration (μM)**	**Amplicon length (bp)**
B5	B5-8584A	III GGG AAA TAG AAT GAT CAG TAC TG***T***	0.1	104
non-B5	B5-8584G	GGA AAT AGA ATG ATC AGT ACT G***C***	0.2	100
	B5-8584F	AAC AAA CCC TGA GAA CCA AAA TGA	0.2	
non-B4c	B4c-1119T	III AGC CCT AAA CCT CAA CAG TTA AA***T***	0.1	94
B4c	B4c-1119C	GCC CTA AAC CTC AAC AGT TAA A***C***	0.2	90
	B4c-1119R	TGA AGC ACC GCC AGG TCC	0.2	
B4a	B4a-9123A	III CGA CAG CGA TTT CTA GGA TAG T***T***	0.2	84
non-B4a	B4a-9123G	GAC AGC GAT TTC TAG GAT AGT ***C***	0.2	80
	B4a-9123F	CAA TAT CAA CCA TTA ACC TTC CCT	0.2	
B4b	B4b-13590A	III CGA GTG CTA TAG GCG CTT GT***T***	0.1	74
non-B4b	B4b-13590G	GAG TGC TAT AGG CGC TTG T***C***	0.2	70
	B4b-13590F	GGA GTC CTA GGC ACA GCT	0.2	
B2	B2-6473T	II TA GGA GAA GTA GGA CTG CTG T***A***	0.2	64
non-B2	B2-6473C	GGA GAA GTA GGA CTG CTG T***G***	0.2	60
	B2-6473F	CCC AAT ACC AAA CGC CCC T	0.2	
non-B4	B4-16217T	III GAT GTG TGA TAG TTG AGG GTT G***A***	0.2	54
B4	B4-16217C	ATG TGT GAT AGT TGA GGG TTG ***G***	0.2	50
	B4-16217F	CCC CCA TGC TTA CAA GCA AG	0.2	
**Multiplex N-VI**				
**Haplogroup**	**Primer name**	**Sequence**	**Concentration (μM)**	**Amplicon length (bp)**
non-F1	F1-10609T	III GGA GTG GGT GTT GAG GGT T***A***	0.1	104
F1	F1-10609C	GAG TGG GTG TTG AGG GTT ***G***	0.2	100
	F1-10609F	TAG TAT ATC GCT CAC ACC TCA TAT C	0.2	
non-F2	F2-1824T	IIII GTA TAG GGG TTA GTC CTT GCT ***A***	0.2	94
F2	F2-1824C	GTA TAG GGG TTA GTC CTT GCT ***G***	0.2	90
	F2-1824F	AAT TGA AAC CTG GCG CAA TAG ATA TAG	0.2	
non-F4	F4-15670T	II TAG CAA TAA TCC CCA TCC TCC A***T***	0.2	84
F4	F4-15670C	GCA ATA ATC CCC ATC CTC CA***C***	0.2	80
	F4-15670R	AGG AGT CAA TAA AGT GAT TGG CTT A	0.2	
F3	F3-10320A	III CAA ACA ACT AAC CTG CCA CTA ATA ***A***	0.2	74
non-F3	F3-10320G	AAA CAA CTA ACC TGC CAC TAA TA***G***	0.2	70
	F3-10320R	GCC AGA CTT AGG GCT AGG AT	0.2	
F1a	F1a-4086T	III GTC AGA AGT AGG GTC TTG GT***A***	0.1	64
non-F1a	F1a-4086C	TCA GAA GTA GGG TCT TGG T***G***	0.2	60
	F1a-4086F	ATG ACG CAC TCT CCC CTG A	0.2	
F1b	F1b-10976T	IIII GCC ATG ATT GTG AGG GGT A***A***	0.2	54
non-F1b	F1b-10976C	GCC ATG ATT GTG AGG GGT A***G***	0.2	50
	F1b-10976F	ACC CCC CTC CTA ATA CTA ACT ACC	0.2	

The letter ‘I’ stands for inosine. Underlined letters indicate non-complementary bases. SNP sites are indicated in bold italics. Haplogroup B is detected by the 9-bp deletion at positions 8281 to 8289.

First of all, reactions using primers of multiplexes M-I and N-I were performed for all samples, because most East-Asian mtDNAs stem from macro-haplogroups M and N, and these multiplexes can identify major branches of macro-haplogroups M and N that are widely observed in East-Asian populations.

Following SNP typing using multiplexes M-I and N-I, each mtDNA under study was classified on the basis of the criteria shown in Fig 2, by using the mtDNA haplogroup nomenclature from Phylotree [19]. Thereafter, each mtDNA underwent subsequent haplogrouping based on the result of multiplexes M-I and N-I. For example, if an mtDNA was designated to haplogroup B, further haplogrouping of this mtDNA was performed using multiplex N-V. If the SNPs observed in a sample did not represent a haplogroup motif, i.e., they were apparently incongruent with Phylotree, the data was discarded because, as we reported previously [29], such incongruence often stems from contamination of the sample.

### PCR conditions and detection of PCR products

The formula of the amplification reaction and the PCR condition were the same for all multiplexes; only the primers differed. Each reaction was performed in a total volume of 10 μl, containing a 1 μl aliquot of the sample DNA solution, optimum concentrations of each primer (Tables 1 and 2), and reagents of the QIAGEN multiplex PCR kit (QIAGEN, Hilden, Germany).

The amplification reaction was conducted in a TaKaRa PCR Thermal Cycler FAST (TaKaRa, Shiga, Japan). The condition for PCR included: incubation at 95°C for 15 minutes; 5 cycles at 94°C for 30 seconds, and at 64°C for 5 minutes (ramp speed > 2.5°C/sec); 33 cycles at 94°C for 30 seconds, and at 64°C for 90 seconds (ramp speed > 2.5°C/sec); and a final extension at 72°C for 3 minutes.

A 2 μl aliquot of the PCR product was separated by electrophoresis in a precast native polyacrylamide gel (10% T, 5% C) containing 1 × TBE buffer with running buffer (1 × TBE) (TEFCO, Tokyo, Japan) using an electrophoretic apparatus STC-808 (TEFCO). The voltage at electrophoresis was 150 V (constant voltage), and the electrophoretic time was approximately 98 minutes. PCR bands were visualized fluorographically after staining with SYBR Green (Bio-Rad Laboratories, Hercules, CA, USA).

### Testing the sensitivity of the new APLP system

To evaluate the sensitivity of our new APLP system, various amounts of crude DNA (1.0 × 10^−9^–0.1 × 10^−15^ g), which included genomic DNA and mtDNA with known haplogroups (D4j and F2), were examined using multiplexes M-I and M-III for D4j mtDNA, and multiplexes N-I and N-VI for F2 mtDNA (Table 1 and Fig 3; Table 2 and Fig 4). The results of the experiments were confirmed by three independent assays. To detect the possibility of contamination, negative PCR controls were also analyzed.

### Application to highly degraded samples

To validate the effectiveness of our new APLP system for highly degraded samples, we analyzed one early Kofun (approximately 1,600 years old) and 11 Middle Jomon (approximately 4,000 years old) skeletons. At first, the ancient DNAs were examined by using multiplexes M-I and N-I. Thereafter, the samples underwent subsequent haplogrouping using multiplexes M-V and N-III. The results of the experiments were confirmed by three independent assays.

Before performing the analysis using our new APLP system, we checked the quality of the ancient DNA samples by using a conventional APLP system [9]; we also checked the direct sequencing of the hypervariable segment I (15999–16366) using our mini-primer sets [29], for which the amplicon length is shorter than 139 bp. The results of the preliminary analyses revealed that 3 out of 12 samples could be assigned to relevant haplogroups using the conventional APLP system: sample B192 assigned to haplogroup N9b, sample B516C to haplogroup M7a, and sample B516D to haplogroup M7a; only one sample (B192), which is ascribed to the early Kofun period, could be analyzed by direct sequencing (mutations identified at the nucleotide positions 15999–16366: A16183C-T16189C-C16223T, relative to rCRS [18]). Along with the ancient samples, negative extraction and negative PCR controls were also analyzed.

## Results

### Hierarchical analysis of mtSNPs

As shown in Figs 3 and 4, our new APLP system correctly identified the genealogy of the mtDNAs for which the haplogroups had been determined in advance.

### Sensitivity of the new APLP system

**Fig 5:**
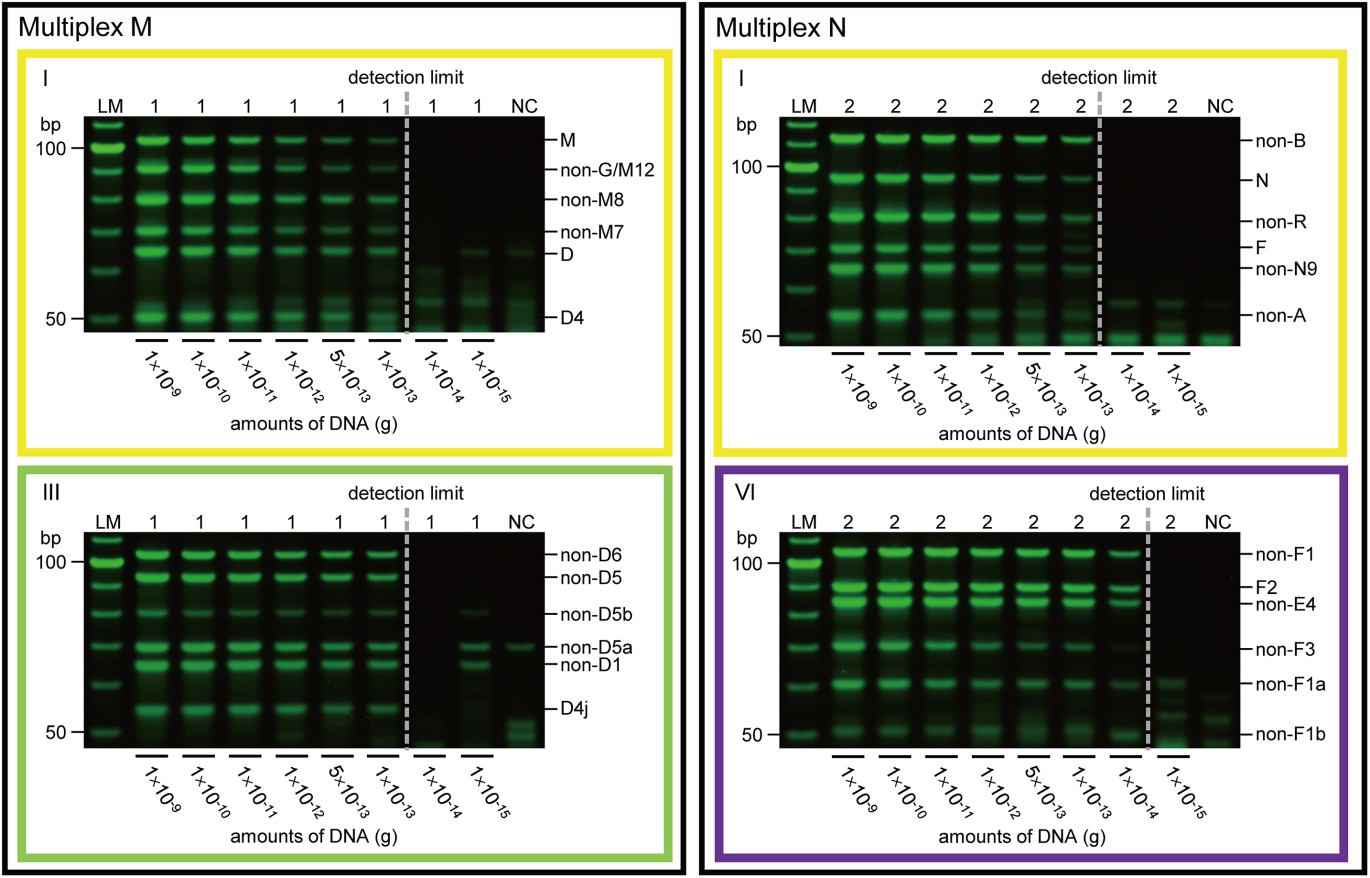
Sensitivity test using progressively diluted crude DNA with known mtDNA haplogroups. Samples 1 and 2 correspond to mtDNA haplogroups D4j and F2, respectively. Results obtained using multiplexes M-I and N-I are framed in yellow, while results obtained using multiplexes M-III and N-VI are framed in light green and purple, respectively. Dotted lines indicate detection limits. NC indicates negative PCR control. LM indicates the 10-bp ladder marker.

Although the copy number of mtDNA exhibits some variation among individuals, the detection limit was 1.0 × 10^−13^ g of crude DNA for multiplexes M-I, M-III, and N-I, and 1.0 × 10^−14^ g for multiplex N-VI. Consequently, our new APLP system correctly identified haplogroups D4j and F2 from 1.0 × 10^−13^ g (100 fg) of crude DNA templates, which corresponds to less than 10 copy numbers of mtDNA (Fig 5). In the analysis of these samples, negative PCR controls were negative throughout the experiment.

### Robustness of the new APLP system with respect to highly degraded mtDNA

**Fig 6:**
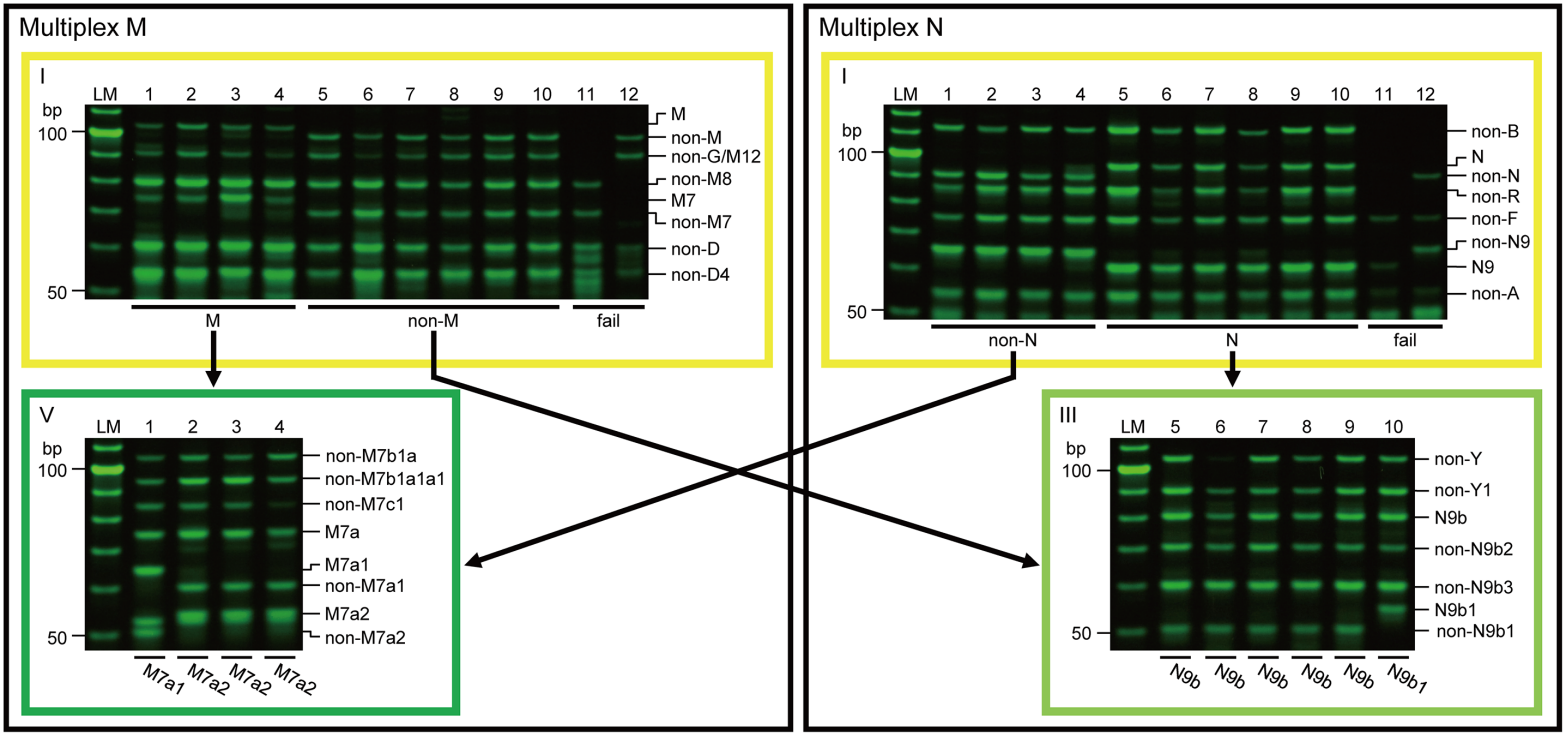
Electrophoretograms of the multiplex PCR products for mtDNA of ancient skeletons. Each lane gives results for a single sample: lane LM, 10-bp ladder marker; lane 1, B516C; lane 2, B516D; lane 3, B202C; lane 4, B228D; lane 5, B192; lane 6, B202A; lane 7, B202B; lane 8, B509A; lane 9, B516A; lane 10, B228C; lane 11, B511; lane 12, B585. Using the conventional APLP system, 3 out of 12 samples could be assigned to relevant haplogroups (B192 to N9b, B516C to M7a, and B516D to M7a). Arrows indicate subsequent haplogrouping flows based on the results obtained using multiplexes M-I and N-I. Yellow frames identify results obtained using multiplexes M-I and N-I, while results obtained using mutiplexes M-V and N-III are framed in green and light green, respectively (color coding corresponds to that given in Fig 2).

By using our new APLP system, 10 out of 12 mtDNAs from the ancient skeletons were successfully assigned to relevant haplogroups (Fig 6). In the analysis of the ancient skeletons, negative extraction and negative PCR controls were negative throughout the experiment (data not shown).

## Discussion

The results showed that, following a hierarchical analysis of 81 haplogroup-defining mtSNPs and 3 insertion/deletion sites performed using fourteen 6-plex multiplexes and subsequent electrophoresis, our new APLP system correctly identified the genealogy of the mtDNAs with known haplogroups. Previously, 15 to 36 mtSNPs and insertion/deletion polymorphisms were examined in East-Asian mtDNA by using conventional means such as SNaPshort minisequencing assays [2, 6, 7] or APLP [5, 9]. However, the number of mtSNPs examined in these previous studies is too small for detailed haplogrouping of East-Asian mtDNAs. In particular, haplogroup D, which is the predominant haplogroup in many East-Asian populations, was not sufficiently classified in the previously reported assays. By using our new APLP system, many major mtDNA lineages including haplogroup D can be securely classified to the sub-haplogroup level.

Moreover, using our new APLP system, hierarchical examination of many mtSNPs can help identify contamination or misinterpretation of the results on the basis of congruence with Phylotree, the updated comprehensive phylogenetic tree of global human mitochondrial DNA variation. Therefore, as we reported previously [29], our new APLP system can improve the reliability of sequencing and SNP analysis of mtDNA.

The recent advent of high throughput sequencing (HTS) technology based on so-called Next Generation Sequencers has allowed analyses of the complete mitochondrial and chromosomal genome sequences even in very degraded samples like those from archaeological skeletons [30, 31]. However, HTS is very costly, and thus it is difficult for most laboratories to perform such analyses routinely. Therefore, to maximize the success rate of HTS, it is important to evaluate the quality and quantity of DNA in the samples before subjecting them to HTS. Our new APLP system correctly identified the haplogroup of mtDNAs in only 100 fg (1.0 × 10^−13^ g) of crude DNA. This sensitivity is over 10 times higher than that reported in previous studies, where quantities of crude DNA in the order of at least pico (1.0 × 10^−12^) grams were required for accurate genotyping [3, 5–7]. This extremely high sensitivity may be ascribable to the reduced number of SNP sites analyzed in each multiplex in our system compared to that analyzed in other systems. Our new APLP system is thus expected to serve as a time- and cost-efficient tool to evaluate the quality and quantity of DNA in samples before HTS analysis.

In the present study, we show how an inosine-flapped APLP system can be efficiently applied for the hierarchical multiplex analysis of mtSNPs. Adding a short inosine extension to the 5'-terminus of APLP primers improves the competitiveness of allele-specific primers to the template DNA, resulting in enhanced reliability of the SNP analysis [28]. Furthermore, the thermodynamics of the primers with inosine flaps have been proven to be less influenced by the sequence of PCR templates than the thermodynamics of the primers with 5' flaps containing ordinary bases [28]. These features of inosine-flapped primers are likely to have contributed to the high sensitivity observed for our new APLP system.

The robustness of our APLP system was verified by the analysis of 12 archaeological skeletons; only 3 such samples could be successfully assigned to relevant haplogroups using the conventional APLP system, whereas a total of 10 samples were successfully assigned using our new APLP system. Our inosine-flapped APLP primers generated shorter amplicons (<110 bp) compared to those generated by conventional APLP primers (<151 bp), and we believe it is the shorter amplicon length that is the source for the higher success rate observed for our new APLP system.

The haplogroups observed in the samples excavated from the Kusakari shell midden site were N9b and M7a. These haplogroups are observed in the Jomon people unearthed from Hokkaido, the northern island of Japan. Notably, haplogroup N9b is the most predominant haplogroup in these Hokkaido Jomon people (64.8%, 35 out of 54 individuals) [13]. The fact that these haplogroups are also observed in the Kusakari Jomon people, who were excavated from Honshu, the main island of Japan, indicates that these haplogroups are strong candidates for the so-called “Jomon genotype” as suggested by the previous studies [9, 12, 13]. Moreover, the fact that haplogroup N9b is observed in the Kofun sample (B192), excavated from the same site, may hint at genetic continuity in this site extending from the Jomon era to the Kofun era.

In addition, at the sub-haplogroup level, one Kusakari Jomon sample (B516C) was assigned to M7a1, which was not observed in the Hokkaido Jomon people [13]. Intriguingly, this sub-haplogroup is the most predominant one found in modern-day Japanese and Korean M7a mtDNAs [22, 32, 33]. It has its highest frequency (44 out of 156 individuals) in Okinawa islanders living in the southern-most islands of Japan [32]. However, haplogroup M7a is rare in Southeast Asian populations, whereas the frequencies of its sister haplogroups (e.g., M7b and M7c) are relatively high in these populations [24, 27, 34]. We have previously hypothesized that haplogroup M7a may have diversified from its ancestral M7 haplogroup in the southern part of the Japanese archipelago [13]. Given the findings here, the fact that haplogroup M7a1 is observed in Honshu Jomon people, but is absent in Hokkaido Jomon individuals, gives some support to this hypothesis.

Unfortunately, we could not compare the robustness of our new system with that of SNaPshot analysis or HTS, mainly because of the residual volume of the samples. However, our system generates short amplicons similar to those reported in the study of Coutinho et al. [6], which focuses on the ancient DNA analysis of the skeletons excavated from South America. Therefore, our system is expected to be as effective as that of Coutinho et al. [6] for the analysis of fragmented mtDNA.

As described earlier, in the case of extremely degraded samples like archaeological skeletons, it is often very difficult to obtain reliable mtDNA sequences. Despite such difficulties, it is worth trying to obtain as much mtDNA data as possible from those samples, because such data is important for phylogeographic analysis, and, in some cases, personal identification. Therefore, our new APLP system is expected to be very useful in analyzing extremely difficult forensic samples, as well as for molecular anthropological studies of ancient populations.
